# Maize Leaf Appearance Rates: A Synthesis From the United States Corn Belt

**DOI:** 10.3389/fpls.2022.872738

**Published:** 2022-04-05

**Authors:** Caio L. dos Santos, Lori J. Abendroth, Jeffrey A. Coulter, Emerson D. Nafziger, Andy Suyker, Jianming Yu, Patrick S. Schnable, Sotirios V. Archontoulis

**Affiliations:** ^1^Department of Agronomy, Iowa State University, Ames, IA, United States; ^2^Cropping Systems and Water Quality Research Unit, USDA-ARS, Columbia, MO, United States; ^3^Department of Agronomy and Plant Genetics, University of Minnesota, St. Paul, MN, United States; ^4^Department of Crop Sciences, College of Agricultural, Consumer and Environmental Sciences, University of Illinois at Urbana–Champaign, Urbana, IL, United States; ^5^School of Natural Resources, University of Nebraska-Lincoln, Lincoln, NE, United States

**Keywords:** phenology, phyllochron, leaf appearance rate, maize, crop models

## Abstract

The relationship between collared leaf number and growing degree days (GDD) is crucial for predicting maize phenology. Biophysical crop models convert GDD accumulation to leaf numbers by using a constant parameter termed phyllochron (°C-day leaf^−1^) or leaf appearance rate (LAR; leaf ^o^C-day^−1^). However, such important parameter values are rarely estimated for modern maize hybrids. To fill this gap, we sourced and analyzed experimental datasets from the United States Corn Belt with the objective to (i) determine phyllochron values for two types of models: linear (1-parameter) and bilinear (3-parameters; phase I and II phyllochron, and transition point) and (ii) explore whether environmental factors such as photoperiod and radiation, and physiological variables such as plant growth rate can explain variability in phyllochron and improve predictability of maize phenology. The datasets included different locations (latitudes between 48° N and 41° N), years (2009–2019), hybrids, and management settings. Results indicated that the bilinear model represented the leaf number vs. GDD relationship more accurately than the linear model (*R*^2^ = 0.99 vs. 0.95, *n* = 4,694). Across datasets, first phase phyllochron, transition leaf number, and second phase phyllochron averaged 57.9 ± 7.5°C-day, 9.8 ± 1.2 leaves, and 30.9 ± 5.7°C-day, respectively. Correlation analysis revealed that radiation from the V3 to the V9 developmental stages had a positive relationship with phyllochron (*r* = 0.69), while photoperiod was positively related to days to flowering or total leaf number (*r* = 0.89). Additionally, a positive nonlinear relationship between maize LAR and plant growth rate was found. Present findings provide important parameter values for calibration and optimization of maize crop models in the United States Corn Belt, as well as new insights to enhance mechanisms in crop models.

## Introduction

The phenological scale for maize (*Zea mays*) development between emergence and the beginning of the reproductive phase is based on successive appearance and collaring of new leaves (Ritchie et al., 1986; Abendroth et al., 2011). Beginning at the first visible collar, developmental stages are defined by the letter V followed by the number of visible collars. For example, the first visible collar would denote the developmental stage V1, while the fifteenth collar would denote the developmental stage V15 (Ritchie et al., 1986; Abendroth et al., 2011). Phenological stages are an important part of managing cropping systems as several crop management decisions depend upon phenology, such as split nitrogen (N) applications (Slaton et al., 2013). Thus, predicting the number of collared leaves accurately in empirical models or complex crop models is decisive (Tollenaar et al., 2018).

The environmental variable influencing phenological development the most is the temperature (Vinocur and Ritchie, 2001). Consequently, crop development is often expressed as a function of cumulative thermal units, specifically growing degree days (GDD; Soltani and Sinclair, 2012). Many different models have been proposed for the accumulation of GDD, including empirical linear, nonlinear, and process-based functions (Kumudini et al., 2014). These functions differ in number and meaning of parameters, and complexity. Process-based models, such as the one developed by Wilson et al. (1995) and used in the APSIM model (Holzworth et al., 2014), offer a level of precision second only to nonlinear empirical models (Kumudini et al., 2014). Process-based functions have the advantage of maintaining their precision when temperatures are greater than the optimum temperature for maize development, demonstrating its usefulness in future scenarios.

Biophysical crop models convert GDD accumulation to leaf numbers by using parameter values termed phyllochron or leaf appearance rate (LAR). While phyllochron is the cumulative thermal time between the appearance of successive leaves in units of °C-day leaf^−1^ (Wilheim and McMaster, 1995), LAR is the reciprocal of phyllochron in units of leaf °C-day^−1^ (Birch et al., 1998). Phyllochron parameter values are crucial for accurately simulating crop growth and development in models such as APSIM (Holzworth et al., 2014) and DSSAT (Hoogenboom et al., 2019). In maize simulation models, once the number of developed leaves reaches its maximum number, crop models trigger flowering, which is a pivotal phenological stage, as stresses during the flowering period can strongly influence maize yield (Bruce et al., 2002; Wang et al., 2019).

In field conditions and with no nutrient or water limitations, phyllochron has been reported as a constant rate from emergence to flowering (Birch et al., 1998), indicating a linear relationship between leaf number and GDD. However, exponential and bilinear relationships have also been utilized in previous research to describe the relationship between leaf number and GDD (Muchow and Carberry, 1989; Abendroth et al., 2011). The bilinear relationship typically has a high phyllochron value at the beginning of the crop’s lifecycle (phase I: slow appearance of leaves) followed by low phyllochron values (phase II: fast appearance of leaves). Common maize phyllochron values for phases I and II are 52 and 36°C-day leaf^−1^ at a base temperature of 8°C (Birch et al., 1998; Van Esbroeck et al., 2008).

Environment, genetics, and management can alter phyllochron values, which can cause inaccuracy in crop model predictions when a constant value is used. For instance, a decrease in radiation has been reported to increase phyllochron (slower appearance of leaves; Birch et al., 1998; Tollenaar, 1999; Tollenaar et al., 2018), while long photoperiods can decrease phyllochron (faster appearance of leaves; Warrington and Kanemasu, 1983). Padilla and Otegui (2005) reported up to 10% variability in phyllochron among 16 maize hybrids and strong coupling between leaf appearance and leaf initiation rate. Van Esbroeck et al. (2008) confirmed the genetic variability in phyllochron in another set of maize hybrids. Muchow and Carberry (1989) and McCullough et al. (1994) reported that water and nitrogen stress can decrease leaf appearance rate. However, the effect of nitrogen stress on leaf appearance is inconsistent across experiments (Vos et al., 2005).

Despite the importance of accurately predicting leaf number and time to flowering, research on maize phyllochron is limited. As a result, most simulation models use phyllochron values developed decades ago. The current literature lacks data for modern maize hybrids and currently we do not know the range of variability that exists in phyllochron to inform crop model parameterization and optimization as well to enable scenarios toward developing future ideotypes (Rötter et al., 2015). For instance, in a comprehensive review of the CERES-Maize model (Jones and Kiniry, 1986) worldwide, Basso et al. (2016) reported a single study that investigated the relationship between leaf number and GDD (Hodges and Evans, 1992). The default phyllochron values in the APSIM classic maize model are 65 (phase I) and 35°C-day leaf ^−1^ (phase II). Extensive APSIM model testing in the United States Corn Belt found that leaf appearance occurs at faster rates of 57 (phase I) and 32°C-day leaf  ^−1^ (phase II; Archontoulis et al., 2014a). Contrastingly, DSSAT works with leaf tips, as opposed to leaf collars, and assumes a constant phyllochron value (Lizaso et al., 2011). The leaf tip method is generally 2.5–5.5 developmental stages ahead of the leaf collar method (Abendroth et al., 2011). The need for research on maize phyllochron is further substantiated by the high turnover rate of maize hybrids in the seed market (Edgerton, 2009).

The current study aims to enhance our knowledge on maize leaf number relative to GDD accumulation by combining and analyzing experimental data from a range of environmental conditions, genotypes, and management settings in the United States Corn Belt. Our first objective is to derive phyllochron parameter values for modern maize hybrids and estimate the range of existing variation in phyllochron values. To do this, we used two frequent used models, simple linear and bilinear. Additionally, we explored environmental and physiological factors that can explain variability in phyllochron. For instance, Tollenaar et al. (2018) proposed adjustments in LAR based on changes in solar radiation. Baumont et al. (2019) identified carbon limitations in wheat LAR, but such a limitation has not been explored in maize. Therefore, our second objective is to explore whether environmental factors such as photoperiod or radiation can explain variability in LAR and improve predictability of maize phenology, and lastly to investigate whether a direct coupling between development and growth exists in maize.

## Materials and Methods

**Figure 1:**
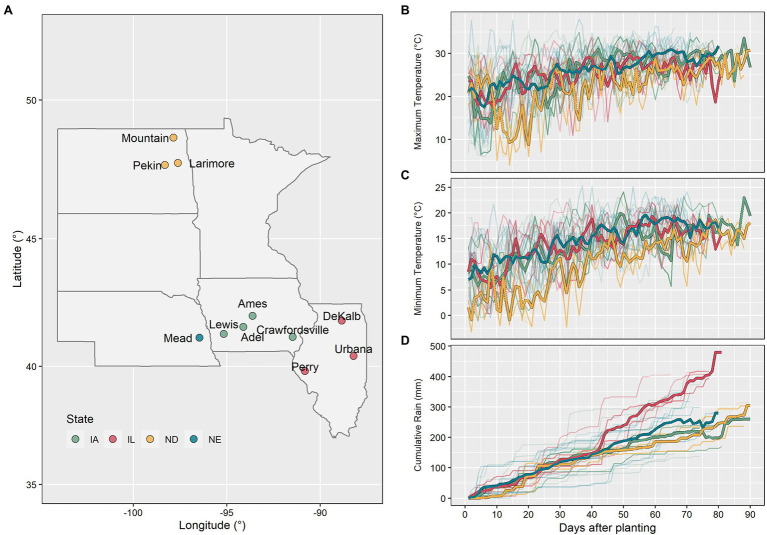
Map with the experimental locations used in this study **(A)**. Maximum temperature **(B)**, minimum temperature **(C)**, and cumulative rain **(D)** between planting and beginning of the reproductive phase for all 98 datasets (thin lines represent individual datasets and thick lines represent the average by state).

We combined 98 datasets with in-season observations of collared leaves from maize experiments in Iowa, Illinois, Nebraska, and North Dakota (Figure 1; Supplementary Table S1). The experiments were replicated, and each dataset had at least five in-season observations during the leaf production phase. In each experiment, leaf numbers were determined based on the V/R system (Ritchie et al., 1986; Abendroth et al., 2011) from emergence until plants reached their maximum leaf number on intervals ranging from 3 to 7 days. Each of the 98 datasets corresponds to a unique combination of location, year (2005–2015), genotype (relative maturity 73–115-day; seed from companies Pioneer, DeKalb, Stine, and Ex-PVP hybrids), and management practices such as previous crop, planting date, irrigation, and nitrogen rate (Supplementary Table S1). The management factors and the hybrids were seldom replicated at different locations and years, limiting our capability to compare the causal effects of genotype, and management practices on LAR. Thus, we analyzed each dataset separately.

**Figure 2:**
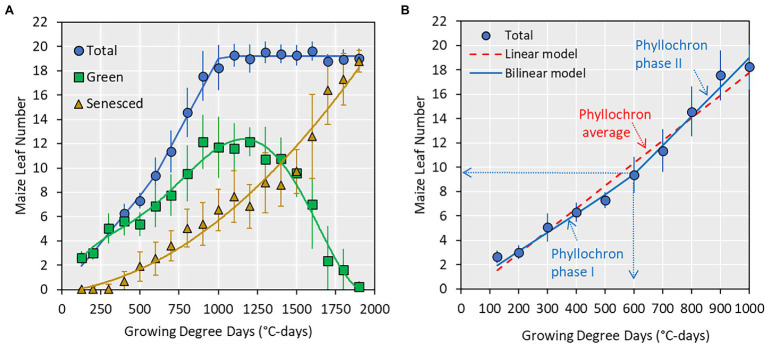
**(A)** Relationship between collared maize leaf number and growing degree days (GDD; base of 8°C). Points indicate average values ± SE from all datasets and lines are best regression fits (not shown). **(B)** A subset of **(A)** showing total leaf number from emergence to silking and the two prediction models used in this study and the associated parameters. The vertical and horizontal dotted arrows indicate the transition point (e.g., 9 leaf number or 600 GDD) at which phyllochron transitions from phase I to phase II.

Daily weather data including minimum and maximum air temperature, precipitation, and solar radiation per site-year were obtained from local weather stations. Daily photoperiod was calculated using the method described by Pereira et al. (2003). The compiled dataset reflected a wide range of environmental conditions (Figure 2). From emergence to flowering, the average minimum air temperature ranged from 12.3 to 20.2°C, the average maximum air temperature from 22.6 to 30.8°C, the average photoperiod from 14.7 to 15.9 h, the cumulative solar radiation from 892.9 to 1485.8 MJm^−2^, and the cumulative precipitation from 95 to 433 mm (Figure 1). The North Dakota sites had the lowest average maximum and minimum air temperatures, while the Iowa sites had the highest.

Beginning at emergence, GDD was calculated as a function of daily average air temperature (Eq. 1; Wilson et al., 1995). We followed the APSIM model approach (Holzworth et al., 2014) in which T_ave_ is the average daily air temperature from eight 3-h interpolations from a third-order polynomial using as inputs minimum and maximum daily temperature.


(1) $$GDD\left(T_{ave},{}^{\circ}C\right)=\left\{\begin{array}{c} 0,T_{ave}\leq 0\\ \frac{T_{ave}}{1.8},0<T_{ave}\leq 18\\ T_{ave}-8,18<T_{ave}\leq 34\\ 26-\left[\left(T_{ave}-34\;\right)\times 2.6\right],34<T_{ave}\leq 44\\ 0,44<T_{ave} \end{array}\right.$$

Data were analyzed in R 4.1 (R Core Team, 2021), and the relationship between GDD and leaf number was investigated by fitting linear and bilinear models. The slope of the linear model represented a single phyllochron value for the entire vegetative period. Conversely, the bilinear model contained a phase I phyllochron value, a transition point, and a phase II phyllochron value (Figure 2). Model fit was assessed by three statistical indices: R-squared, modeling efficiency, and relative root mean square error (equations in Archontoulis and Miguez, 2015).

The relationships between model coefficients and environmental variables were investigated in a correlation analysis at different windows after emergence. Average air temperature, photoperiod, radiation, and cumulative precipitation were calculated at different windows between emergence and flowering for each dataset. The examined window began at emergence and increased in 26°C-day intervals (equivalent to 1 biological day) until flowering. For instance, the environmental variables were calculated between 0 and 26°C-day, 0 and 52°C-day, and so on. Then, the beginning of the window was advanced to 26°C-day and the same process was applied. This process was repeated for all subsequent combinations of the beginning and end of the window. A similar search approach was followed by Li et al. (2018) and Guo et al. (2020).

In five out of the 98 total datasets (dataset ID from 6 to 10 in Supplementary Table S1), we had detailed information on in-season biomass accumulation, and leaf number (Archontoulis et al., 2020). Each of these random variables were fit to regression exponential models (see Supplementary Materials for the goodness of fit) and the predicted values of these models were resampled every 100°C-day to obtain point values of plant growth rate and LAR. The relationship between instant LAR and instant plant growth rate was investigated using nonlinear Michaelis–Menten models (Archontoulis and Miguez, 2015).

## Results

Across all datasets, the average maximum leaf number was 20 leaves (Figure 2, range 16–23, Supplementary Table S1). The appearance of collared leaves followed a bilinear pattern between emergence and flowering. The maize plant was able to maintain a maximum of 14 green leaves during the vegetative period, with loss of lower canopy leaves beginning at approximately the V6 stage (Figure 2). The leaf senescence followed an exponential pattern until physiological maturity. In this study, we use the total leaf number from emergence to flowering (commensurate with the V-stage) and fit two types of models (linear and bilinear; Figure 2) to derive phyllochron parameters.

**Figure 3:**
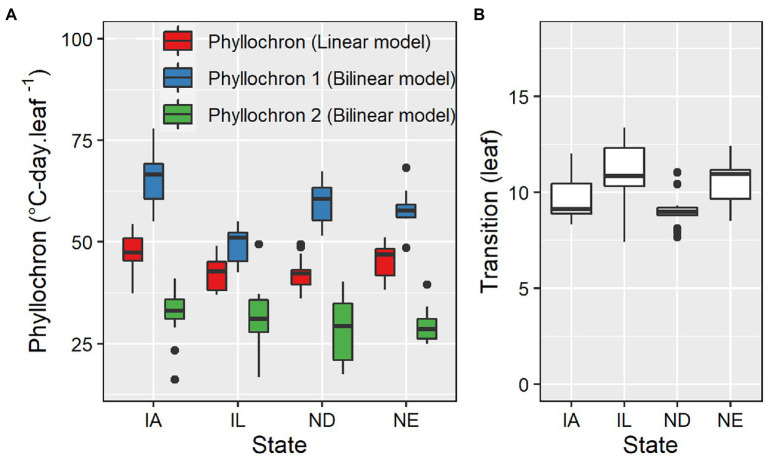
Phyllochron parameter values for linear and bilinear models **(A)** and transition leaf number for the bilinear model **(B)** for datasets in IA (*n* = 64), IL (*n* = 13), ND (*n* = 13), and NE (*n* = 8).

Phyllochron determined by linear models ranged from 36.1 to 54.8°C-day leaf^−1^, averaging 51.5°C-day leaf^−1^ (Figure 3). The coefficient of variation was 9.4%. The first and second phase phyllochron values in the bilinear models ranged from 42.5 to 77.9°C-day leaf^−1^ and from 16.2 to 49.5°C-day leaf^−1^, averaging 57.9°C-day leaf^−1^ and 30.9°C-day leaf^−1^, respectively (Figure 3). The transition point between the two phases of the bilinear model ranged from 7.4 to 13.4 leaves, averaging 9.8 leaves (Figure 3). The phyllochron values and the obtained variability in phyllochron were consistent among locations even though each location included a different set of management factors and hybrids (Supplementary Table S1). The coefficient of variation for the phyllochron I, phyllochron II, and the transition point observed across 98 datasets was 13, 19, and 12%, respectively.

**Figure 4:**
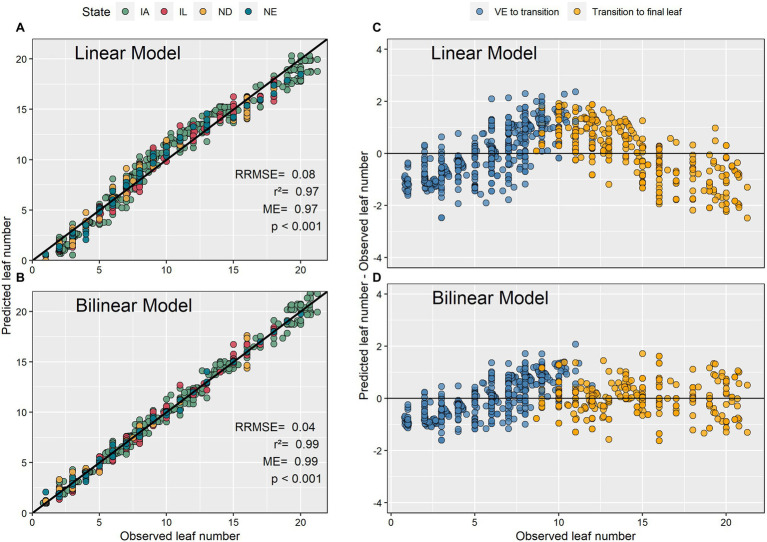
Predicted and observed maize leaf numbers using linear and bilinear models **(A,B)** and their associated residual plots **(C,D)**.

The bilinear model estimated V-stages (GDD accumulation) more accurately than the linear model (Figure 4). The bilinear model had a 3% higher modeling efficiency, a 40% lower relative root mean square error, and a lower bias compared to the linear model. The residual plots showed that the linear model overpredicted GDD at the beginning and the end of the vegetative period. The model residuals ranged from −2 to 2 leaves (Figure 4).

**Figure 5:**
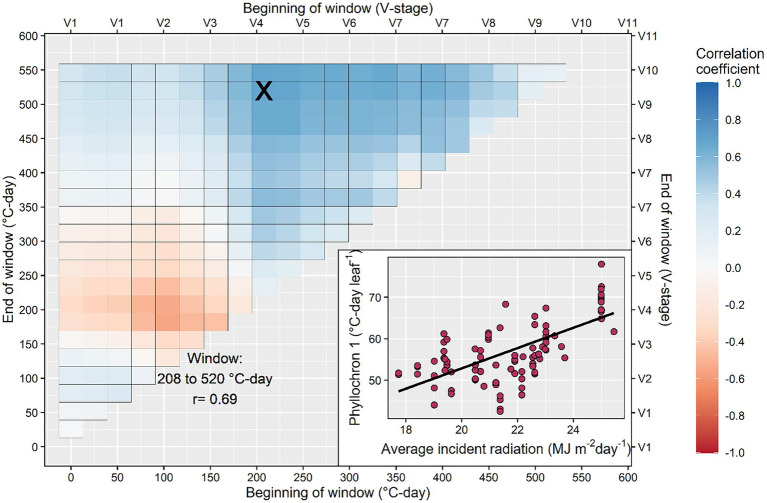
Search process to identify the critical window during the growing season in which average incident radiation determines the rate of leaf appearance. The inset figure shows in detail the strongest relationship between phyllochron and average incident radiation calculated in the interval between 208 and 520°C-day. This interval corresponds to the period between the 4th and the 10th collared leaf, approximately.

The search for correlation between model coefficients (phyllochron values and transition point) and environmental variables produced inconsistent results (Figure 5; Supplementary Figures S1**–**S4). The average radiation calculated between 208 and 520°C-day had a positive correlation with the phase I phyllochron of the bilinear model (*r* = 0.69; Figure 5), suggesting that high radiation will slow initial leaf appearance. Further, the average radiation from 182 to 546°C-day had a significant negative relationship with the transition leaf in the bilinear model (*r* = −0.52; Supplementary Figure S1) suggesting that high radiation will accelerate the transition from phase I to II phyllochron. Weak correlations were found between radiation and the phyllochron of the linear model or phase II phyllochron of the bilinear model (Supplementary Figure S1). Photoperiod had a weak correlation with phyllochron but a strong positive correlation with time to flowering (Supplementary Figure S2). The average temperature calculated between the windows 156–208°C-day and 130–234°C-day presented an inverse relationship with phyllochron values of the linear model (*r* = −0.5) and the phase I phyllochron of the bilinear model (*r* = −0.66; Supplementary Figure S3). The cumulative precipitation calculated in the window 234–338°C-day presented an inverse relationship with the phase I phyllochron of the bilinear model (*r* = −0.54; Supplementary Figure S4), suggesting that water-limited conditions (reduced prediction) slow leaf appearance.

**Figure 6:**
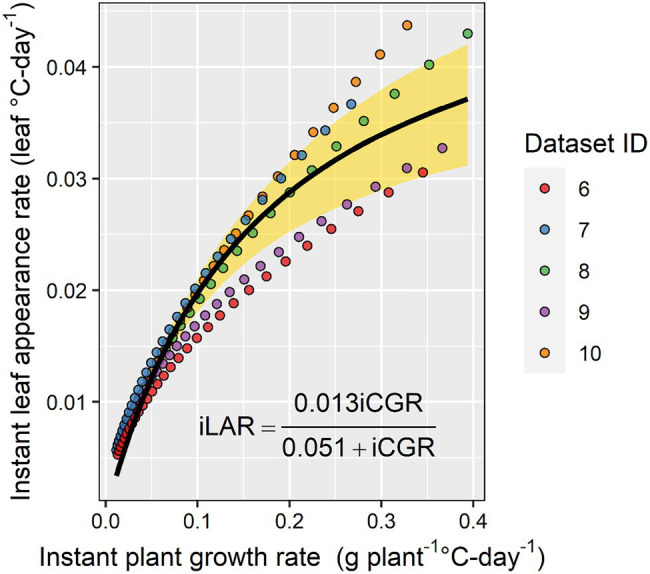
The relationship between instant leaf appearance rate and instant crop growth rate. The yellow shaded area represents the 95% CI.

In a subset of the experimental datasets with the detailed in-season biomass observations, the relationship between instant LAR and instant plant growth rate was positive and characterized by a rectangular hyperbola relationship (Figure 6). As the instant plant growth rate increased, the instant LAR increased, but as the instant plant growth rate continued to increase beyond 0.3 g plant^−1^ GDD^−1^, the instant LAR did not increase at the same rate (Figure 6).

## Discussion

The present study analyzed 98 recent experimental datasets to advance our predictive capabilities and knowledge pertaining to the maize leaf number-GDD relationship. This is important as many crop management decisions are phenology dependent. Between differing methodologies of leaf tips and collared leaves, we focused on collared leaves because leaf collars are most frequently used to determine maize phenology (Ritchie et al., 1986; Abendroth et al., 2011). Our findings indicated that the use of a linear model to describe the leaf number-GDD relationship suffers from systemic over and underestimations, especially around the middle of the V phase (Figure 4) and should be used with caution. The bilinear model described the relationship between leaf number and GDD with greater accuracy. These results are consistent with previous research that reported an acceleration in leaf collaring after the V8 to V11 developmental stages (Warrington and Kanemasu, 1983; Zhu et al., 2014). Furthermore, Ritchie and NeSmith (1991) argued that leaf collaring in plants with large leaves occurs more rapidly for the last few leaves as a function of an accelerated expansion of the internodes, when compared to the beginning to the developmental cycle. Similarly, Warrington and Kanemasu (1983) observed an acceleration in leaf appearance rate after the V12 developmental stage and associated this to rapid stem elongation. We theorize that the bilinear model represents the relationship between leaf number and GDD more accurately than linear models by capturing the acceleration in leaf collaring expansion resulting from rapid internode expansion. The use of bilinear models is advised in future studies.

Limitations of our datasets did not allow us to delineate the effect of genotypes or management practices on maize phyllochron. However, our summary analysis provided valuable insights, especially on the range of phyllochron values in modern maize hybrids and correlations with environmental and physiological factors. The range of phyllochron values is pivotal for crop model calibration, and the development of parameter values within physiological limits for optimization (Jones et al., 2011; Archontoulis et al., 2014b). This range of parameter values is also needed for scenario studies and ideotype design. The present study has identified a range of values for phyllochron I (36.1–54.77°C-day leaf^−1^), phyllochron II (16.2–49.5°C-day leaf^−1^), and transition point (7.4–13.4 leaves). The values agree well with previous estimates using the same base temperature for GDD accumulation (Birch et al., 1998; Van Esbroeck et al., 2008; Archontoulis et al., 2014a). Caution should be exercised when interpreting literature values as the base temperature and model used affect the magnitude of phyllochron estimates (Padilla and Otegui, 2005).

Across a range of different locations, hybrids, and management practices, we estimated a 13% variability in phyllochron I, a 19% variability in phyllochron II and a 12% variability in the transition point. The observed variability in our study is higher than the 10% variability observed by Padilla and Otegui (2005) in a study exploring 16 maize hybrids. To put that in perspective, a 10% change in phyllochron I and II values can change flowering time by 5 days in central Iowa, United States. Therefore, the observed 13–19% coefficient of variation in phyllochron can alter flowering time by over a week. This can have large consequences in crop models because phyllochron affects plant processes such as leaf area index, biomass partitioning, N uptake, and therefore grain yield. This reinforces the need for accurate estimation of phyllochron to accurately predict leaf number and therefore maize phenology.

Our results indicate the average solar incident radiation between 208 and 520°C-day (roughly, between the V3 and V9 developmental stages) had a positive correlation with the phase I phyllochron (Figure 5). This suggests leaf appearance decelerates as radiation increases, contrasting previous research that indicated a faster leaf appearance as radiation increased (Birch et al., 1998; Tollenaar et al., 2018). Part of this discrepancy may be due to our experimental dataset, which contained a range of genotypes and management settings across temperate environments as opposed to a single factor-location controlled experiment. Another reason may be the period considered for the correlation analysis and the type of model used (linear vs. bilinear). More research is needed in this area. We explored all possible combinations of periods, similarly to Guo et al. (2020) for rice and Li et al. (2018) for sorghum, with the result of V3–V9 developmental stages as the most important. Tollenaar et al. (2018) used the previous week’s radiation to adjust phyllochron.

Although previous studies have shown an influence of photoperiods greater than 13 h in leaf appearance rate (Warrington and Kanemasu, 1983), photoperiod did not explain the variability in phyllochron in our analysis. Warrington and Kanemasu (1983) investigated the effect of photoperiod in leaf appearance rate at 18 and 28°C in a controlled environment room and found the effect of photoperiod was present only in the lower temperature regime. In our study, we rarely encountered consecutive days with low temperatures and long days. Additionally, we found that photoperiod was positively correlated with total leaf number (time to flowering) which agrees with previous findings (Tollenaar et al., 2018). Similarly, the average temperature between 0 and 338°C-day presented a strong (*r* = −089; Supplementary Figure S3) negative relationship with the number of days to reach flowering which agrees with research by Guo et al. (2020). However, we were not able to confirm the correlation between phyllochron and average temperature reported by Birch et al. (1998).

Baumont et al. (2019) have shown that phyllochron can be limited by carbon availability in wheat. In the present study, we provided evidence that the carbon limitation theory also holds for maize (Figure 6). However, our findings are based on in-season estimates and not on whole season estimates as done by Baumont et al. (2019). This topic deserves further research as direct linkages between plant development and growth can stimulate further enhancements in mechanistic crop modeling, i.e., reduce the number of input parameters and empiricism in models. Currently, in crop modeling, development has a substantial influence on growth, but growth has very little influence on development.

## Conclusion

Our research advanced the leaf number-GDD mathematical relationship and for the first time developed a range of phyllochron values for modern maize hybrids growing across a range of management settings in the United States Corn Belt (98 datasets). The present results can improve the predictability of leaf number, an important attribute for timely crop management, and can assist crop model optimization and scenario tasks. We also identified correlations between phyllochron and radiation, and plant growth rate that can stimulate model improvements. As maize hybrids continue to rapidly change in the market, research on the leaf number–GDD relationship should be regularly updated given the importance of accurately predicting phenology.

## Data Availability Statement

The raw data supporting the conclusions of this article will be made available by the authors, without undue reservation.

## Author Contributions

CdS and SA designed the study. SA, JC, LA, AS, and EN contributed datasets. CdS performed data analysis. All authors contributed to the article and approved the submitted version.

## Funding

This work was sponsored by NSF (#1830478, #1842097), USDA Hatch project (IOW10480), the Iowa State University Plant Sciences Institute, Stine Seed, and Pioneer Crop Management research award.

## Conflict of Interest

PS is a co-lead of the Genomes to Fields Initiative and PI of the USDA-NIFA funded Agricultural Genome to Phenome Initiative. He is co-founder of Data2Bio, LLC; Dryland Genetics, Inc.; EnGeniousAg, LLC; and LookAhead Breeding, LLC. He is a member of the scientific advisory board and a shareholder of Hi-Fidelity Genetics, Inc., and a member of the scientific advisory boards of Kemin Industries and Centro de Tecnologia Canavieira. He is a recipient of research funding from Iowa Corn and Bayer Crop Science.

The remaining authors declare that the research was conducted in the absence of any commercial or financial relationships that could be construed as a potential conflict of interest.

## Publisher’s Note

All claims expressed in this article are solely those of the authors and do not necessarily represent those of their affiliated organizations, or those of the publisher, the editors and the reviewers. Any product that may be evaluated in this article, or claim that may be made by its manufacturer, is not guaranteed or endorsed by the publisher.
