# Supplementary material for: Maize Leaf Appearance Rates: A Synthesis From the United States Corn Belt
Source: Front Plant Sci. 2022 Apr 5;13:872738. doi: 10.3389/fpls.2022.872738 (PMC9037294; doi:10.3389/fpls.2022.872738)
Supplement: Supplementary file 1 [file Table_1.docx]

| **Table S1**. Overview of the experimental datasets. Data are grouped by study (e.g., datasets 1–6 refer to different hybrids studied in a single location/year). RMG refers to relative maturity group, GDD to R1 refers to cumulative thermal time to reach silking (R1 stage), LN is the final leaf number (including standard error), Phyllochron Linear is the phyllochron estimated by the linear model, Phyllochron Phase 1 is the phyllochron estimated first phase phyllochron estimated by the bilinear model, Phyllochron Phase 2 is the phyllochron estimated second phase phyllochron estimated by the bilinear model, Transition is the transition between phases 1 and 2 of the phyllochron estimated by the bilinear model.  All data collected under rainfed conditions except datasets ID: 92, 95, and 98. All data collected under non-limited N fertilizer except datasets ID: 11 and 13. Previous crop was always maize except datesets ID 11 and 12. | | | | | | | | | | | |
| --- | --- | --- | --- | --- | --- | --- | --- | --- | --- | --- | --- |
| ID | Group | Location | Planting | Emergence | RMG | GDD to R1 | LN | Phyllochron Linear | Phyllochron Phase 1 | Phyllochron Phase 2 | Transition |
|  |  |  |  |  | day | °C-day | leaf | °C-day leaf^-1^ | °C-day leaf^-1^ | °C-day leaf^-1^ | leaf |
| 1 | Hybrid  X  Relative Maturity | Ames, IA | 2009-05-19 | 2009-05-24 | 114 | 975 | 21.0±0.1 | 46.09 | 60.58 | 25.82 | 11.08 |
| 2 |  | Ames, IA | 2009-05-19 | 2009-05-24 | 104 | 975 | 21.0±0.1 | 46.46 | 59.82 | 28.95 | 10.73 |
| 3 |  | Ames, IA | 2009-05-19 | 2009-05-24 | 110 | 975 | 20.0±0.1 | 48.71 | 60.41 | 28.42 | 11.19 |
| 4 |  | Ames, IA | 2009-05-19 | 2009-05-24 | 112 | 975 | 20.0±0.2 | 49.73 | 61.38 | 27.14 | 11.34 |
| 5 |  | Ames, IA | 2009-05-19 | 2009-05-24 | 113 | 975 | 19.0±0.2 | 49.03 | 60.93 | 32.86 | 10.20 |
| 6 |  | Ames, IA | 2015-05-21 | 2015-05-29 | 111 | 975 | 19.0±0.0 | 37.39 | 57.55 | 16.22 | 10.53 |
| 7 | Location  x  Hybrid | Lewis, IA | 2016-04-24 | 2016-05-12 | 111 | 987 | 17.0±0.3 | 45.02 | 55.13 | 23.26 | 12.03 |
| 8 |  | Ames, IA | 2017-04-25 | 2017-05-11 | 113 | 1102 | 18.0±0.0 | 45.53 | 61.69 | 35.08 | 8.99 |
| 9 |  | Crawfordsville, IA | 2017-05-16 | 2017-05-31 | 113 | - | 17.0±0.0 | 50.07 | 55.16 | 38.25 | 10.72 |
| 10 |  | Lewis, IA | 2017-05-05 | 2017-05-15 | 115 | 1046 | 20.0±0.6 | 39.73 | 60.71 | 30.34 | 8.55 |
| 11 | Rotation  x  Nitrogen | Ames, IA | 2020-04-22 | 2020-05-02 | 108 | 954 | 19.3±0.3 | 47.83 | 77.97 | 29.72 | 10.50 |
| 12 |  | Ames, IA | 2020-04-22 | 2020-05-02 | 108 | 1065 | 20.3±0.3 | 43.16 | 72.54 | 38.78 | 8.32 |
| 13 |  | Ames, IA | 2020-04-22 | 2020-05-02 | 108 | 1065 | 20.7±0.3 | 45.84 | 64.81 | 39.19 | 10.38 |
| 14 |  | Ames, IA | 2020-04-22 | 2020-05-02 | 108 | 1065 | 20.7±0.3 | 43.70 | 66.83 | 39.78 | 8.98 |
| 15 | Year 1 Planting Date x  Relative Maturity | Ames, IA | 2010-04-30 | 2010-05-14 | 93 | 700 | 18.6±0.2 | 46.78 | 59.19 | 22.99 | 9.85 |
| 16 |  | Ames, IA | 2010-04-30 | 2010-05-14 | 98 | 715 | 19.1±0.3 | 42.92 | 56.96 | 28.17 | 9.27 |
| 17 |  | Ames, IA | 2010-04-30 | 2010-05-14 | 105 | 807 | 20.5±0.2 | 41.64 | 55.32 | 21.48 | 11.00 |
| 18 |  | Ames, IA | 2010-04-30 | 2010-05-14 | 112 | 821 | 19.6±0.2 | 48.18 | 61.18 | 26.70 | 10.00 |
| 19 |  | Ames, IA | 2010-05-17 | 2010-05-24 | 93 | 689 | 18.4±0.2 | 44.06 | 55.55 | 27.26 | 9.01 |
| 20 |  | Ames, IA | 2010-05-17 | 2010-05-24 | 98 | 704 | 19.3±0.2 | 43.32 | 52.09 | 26.84 | 9.76 |
| 21 |  | Ames, IA | 2010-05-17 | 2010-05-24 | 105 | 785 | 20.1±0.2 | 39.17 | 52.12 | 26.43 | 10.00 |
| 22 |  | Ames, IA | 2010-05-17 | 2010-05-24 | 112 | 819 | 18.5±0.2 | 48.39 | 57.13 | 28.41 | 10.33 |
| 23 |  | Ames, IA | 2010-05-28 | 2010-06-02 | 93 | 748 | 17.9±0.2 | 49.13 | 54.54 | 32.72 | 9.43 |
| 24 |  | Ames, IA | 2010-05-28 | 2010-06-02 | 98 | 748 | 18.8±0.2 | 46.48 | 51.57 | 29.57 | 10.99 |
| 25 |  | Ames, IA | 2010-05-28 | 2010-06-02 | 105 | 782 | 20.1±0.2 | 44.19 | 54.93 | 28.45 | 9.31 |
| 26 |  | Ames, IA | 2010-05-28 | 2010-06-02 | 112 | 797 | 18.8±0.2 | 47.61 | 55.97 | 31.17 | 10.18 |
| 27 |  | Ames, IA | 2010-06-11 | 2010-06-15 | 93 | 722 | 17.8±0.0 | 44.30 | 55.53 | 24.54 | 9.71 |
| 28 |  | Ames, IA | 2010-06-11 | 2010-06-15 | 98 | 738 | 18.7±0.0 | 45.04 | 54.01 | 29.70 | 9.93 |
| 29 |  | Ames, IA | 2010-06-11 | 2010-06-15 | 105 | 774 | 19.5±0.0 | 41.94 | 53.37 | 27.44 | 10.00 |
| 30 |  | Ames, IA | 2010-06-11 | 2010-06-15 | 112 | 756 | 18.4±0.0 | 46.14 | 57.77 | 31.54 | 9.28 |
| 31 |  | Ames, IA | 2010-06-25 | 2010-07-01 | 93 | 672 | 17.7±0.0 | 42.39 | 50.31 | 26.50 | 9.59 |
| 32 |  | Ames, IA | 2010-06-25 | 2010-07-01 | 98 | 698 | 18.4±0.0 | 44.76 | 48.11 | 35.14 | 9.87 |
| 33 |  | Ames, IA | 2010-06-25 | 2010-07-01 | 105 | 731 | 19.3±0.0 | 44.40 | 46.43 | 31.17 | 11.01 |
| 34 |  | Ames, IA | 2010-06-25 | 2010-07-01 | 112 | 758 | 17.9±0.0 | 46.34 | 52.06 | 33.06 | 10.00 |
| 35 | Year 2 Planting Date  X  Relative Maturity | Ames, IA | 2011-05-03 | 2011-05-12 | 93 | 747 | 18.4±0.2 | 48.53 | 53.23 | 29.12 | 9.91 |
| 36 |  | Ames, IA | 2011-05-17 | 2011-05-25 | 93 | 731 | 18.3±0.2 | 46.76 | 53.65 | 28.62 | 9.76 |
| 37 |  | Ames, IA | 2011-05-31 | 2011-06-06 | 93 | 727 | 18.4±0.6 | 48.75 | 50.41 | 35.93 | 9.68 |
| 38 |  | Ames, IA | 2011-06-08 | 2011-06-16 | 93 | 712 | 18.3±0.4 | 44.07 | 49.65 | 33.66 | 9.75 |
| 39 |  | Ames, IA | 2011-05-03 | 2011-05-12 | 98 | 747 | 19.3±0.1 | 47.50 | 51.51 | 28.64 | 10.35 |
| 40 |  | Ames, IA | 2011-05-17 | 2011-05-25 | 98 | 764 | 19.3±0.4 | 45.01 | 52.45 | 31.47 | 9.79 |
| 41 |  | Ames, IA | 2011-05-31 | 2011-06-06 | 98 | 746 | 19.1±0.3 | 48.32 | 48.90 | 32.72 | 11.51 |
| 42 |  | Ames, IA | 2011-06-08 | 2011-06-16 | 98 | 712 | 19.1±0.6 | 43.81 | 48.73 | 32.38 | 10.22 |
| 43 |  | Ames, IA | 2011-06-24 | 2011-06-30 | 98 | 685 | 18.3±0.5 | 47.95 | 47.49 | 38.82 | 11.17 |
| 44 |  | Ames, IA | 2011-05-03 | 2011-05-12 | 105 | 799 | 20.4±0.2 | 48.69 | 53.46 | 29.57 | 10.00 |
| 45 |  | Ames, IA | 2011-05-17 | 2011-05-25 | 105 | 846 | 23.3±0.6 | 44.07 | 54.64 | 28.67 | 10.32 |
| 46 |  | Ames, IA | 2011-05-31 | 2011-06-06 | 105 | 795 | 20.1±0.3 | 42.81 | 49.35 | 22.72 | 12.22 |
| 47 |  | Ames, IA | 2011-06-08 | 2011-06-16 | 105 | 731 | 19.4±0.8 | 38.48 | 50.12 | 20.44 | 11.48 |
| 48 |  | Ames, IA | 2011-06-24 | 2011-06-30 | 105 | 711 | 19.5±0.2 | 43.57 | 46.56 | 32.89 | 11.34 |
| 49 |  | Ames, IA | 2011-05-17 | 2011-05-25 | 112 | 816 | 19.3±0.5 | 46.26 | 60.43 | 32.20 | 8.64 |
| 50 |  | Ames, IA | 2011-05-31 | 2011-06-06 | 112 | 808 | 19.7±0.3 | 47.11 | 53.76 | 29.98 | 10.72 |
| 51 |  | Ames, IA | 2011-06-08 | 2011-06-16 | 112 | 731 | 19.2±0.7 | 45.57 | 53.61 | 34.34 | 9.22 |
| 52 |  | Ames, IA | 2011-06-24 | 2011-06-30 | 112 | 759 | 18.8±0.3 | 49.18 | 51.98 | 39.65 | 10.04 |
| 53 | Hybrid  X  Planting Arrangement | Adel, IA | 2017-04-24 | 2017-05-05 | 107 | - | 18.3±0.0 | 51.76 | 70.29 | 36.46 | 8.62 |
| 54 |  | Adel, IA | 2017-04-24 | 2017-05-05 | 107 | - | 18.3±0.3 | 53.81 | 68.87 | 36.29 | 9.02 |
| 55 |  | Adel, IA | 2017-04-24 | 2017-05-05 | 107 | - | 18.3±0.3 | 53.01 | 69.45 | 36.46 | 8.84 |
| 56 |  | Adel, IA | 2017-04-24 | 2017-05-05 | 107 | - | 18.0±0.3 | 50.70 | 70.08 | 32.49 | 8.87 |
| 57 |  | Adel, IA | 2017-04-24 | 2017-05-05 | 107 | - | 19.7±0.3 | 47.52 | 66.84 | 32.39 | 8.77 |
| 58 |  | Adel, IA | 2017-04-24 | 2017-05-05 | 107 | - | 19.7±0.3 | 47.62 | 67.14 | 32.39 | 8.70 |
| 59 |  | Adel, IA | 2017-04-24 | 2017-05-05 | 107 | - | 19.7±0.3 | 47.52 | 66.84 | 32.39 | 8.77 |
| 60 |  | Adel, IA | 2017-04-24 | 2017-05-05 | 107 | - | 18.7±0.3 | 51.15 | 65.88 | 29.94 | 10.00 |
| 61 |  | Adel, IA | 2017-04-24 | 2017-05-05 | 107 | - | 19.0±0.3 | 52.90 | 69.69 | 31.61 | 9.28 |
| 62 |  | Adel, IA | 2017-04-24 | 2017-05-05 | 107 | - | 19.0±0.3 | 51.87 | 72.39 | 33.50 | 8.42 |
| 63 |  | Adel, IA | 2017-04-24 | 2017-05-05 | 107 | - | 19.0±0.3 | 53.72 | 68.40 | 31.61 | 9.61 |
| 64 |  | Adel, IA | 2017-04-24 | 2017-05-05 | 107 | - | 18.7±0.0 | 54.77 | 70.44 | 35.86 | 8.76 |
| 65 | Hybrid  x  Planting Date  x  Location | Dekalb, IL | 2015-05-02 | 2015-05-20 | 103 | - | - | 42.73 | 54.58 | 16.75 | 11.40 |
| 66 |  | Dekalb, IL | 2015-05-02 | 2015-05-20 | 108 | - | - | 42.58 | 51.09 | 18.29 | 12.30 |
| 67 |  | Dekalb, IL | 2015-05-02 | 2015-05-20 | 113 | - | - | 45.51 | 48.15 | 24.39 | 13.39 |
| 68 |  | Dekalb, IL | 2015-05-02 | 2015-05-20 | 93 | - | 16.0±0.0 | 47.88 | 44.01 | 49.47 | 10.86 |
| 69 |  | Perry, IL | 2015-04-21 | 2015-05-10 | 109 | - | 19.0±0.0 | 44.23 | 51.36 | 37.11 | 10.31 |
| 70 |  | Perry, IL | 2015-04-21 | 2015-05-10 | 114 | - | 19.0±0.0 | 49.02 | 51.71 | 34.95 | 13.06 |
| 71 |  | Urbana, IL | 2015-04-17 | 2015-05-10 | 103 | 725 | 19.0±0.0 | 38.35 | 43.03 | 35.71 | 10.86 |
| 72 |  | Urbana, IL | 2015-04-17 | 2015-05-10 | 108 | 725 | 20.0±0.3 | 38.16 | 45.27 | 30.08 | 11.28 |
| 73 |  | Urbana, IL | 2015-04-17 | 2015-05-10 | 113 | 815 | 19.0±0.0 | 37.22 | 46.21 | 31.10 | 10.50 |
| 74 |  | Urbana, IL | 2015-04-17 | 2015-05-10 | 93 | 725 | 19.0±0.2 | 37.26 | 42.46 | 27.83 | 12.34 |
| 75 |  | Urbana, IL | 2015-05-14 | 2015-05-25 | 109 | 784 | 19.0±0.0 | 43.07 | 52.34 | 37.23 | 7.42 |
| 76 |  | Urbana, IL | 2015-04-18 | 2015-05-08 | 112 | 857 | 19.0±0.0 | 36.99 | 52.66 | 31.14 | 9.20 |
| 77 |  | Urbana, IL | 2015-05-14 | 2015-05-25 | 112 | 784 | - | 45.20 | 55.08 | 30.50 | 8.33 |
| 78 | Hybrid  X  Location | Larimore, ND | 2015-04-29 | 2015-05-25 | 73 | - | - | 48.73 | 60.59 | 31.01 | 9.30 |
| 79 |  | Larimore, ND | 2015-04-29 | 2015-05-25 | 76 | - | - | 42.21 | 67.37 | 24.41 | 7.94 |
| 80 |  | Larimore, ND | 2015-04-29 | 2015-05-25 | 79 | - | - | 47.18 | 57.28 | 40.30 | 7.65 |
| 81 |  | Larimore, ND | 2015-04-29 | 2015-05-25 | 82 | - | - | 42.42 | 63.11 | 24.41 | 8.81 |
| 82 |  | Larimore, ND | 2015-04-29 | 2015-05-25 | 85 | - | - | 43.15 | 59.08 | 29.30 | 8.83 |
| 83 |  | Larimore, ND | 2015-04-29 | 2015-05-25 | 86 | - | - | 42.79 | 61.66 | 29.30 | 8.12 |
| 84 |  | Mountain, ND | 2015-04-25 | 2015-05-20 | 73 | - | - | 36.10 | 51.53 | 20.91 | 11.03 |
| 85 |  | Mountain, ND | 2015-04-25 | 2015-05-20 | 76 | - | - | 39.77 | 55.99 | 33.02 | 7.99 |
| 86 |  | Mountain, ND | 2015-04-25 | 2015-05-20 | 79 | - | - | 39.50 | 51.87 | 34.85 | 9.21 |
| 87 |  | Mountain, ND | 2015-04-25 | 2015-05-20 | 82 | - | - | 36.49 | 63.41 | 20.91 | 8.93 |
| 88 |  | Mountain, ND | 2015-04-25 | 2015-05-20 | 85 | - | - | 41.94 | 65.42 | 20.91 | 9.00 |
| 89 |  | Mountain, ND | 2015-04-25 | 2015-05-20 | 86 | - | - | 38.18 | 65.42 | 17.42 | 9.07 |
| 90 |  | Pekin, ND | 2015-04-29 | 2015-05-25 | 79 | - | - | 49.49 | 55.38 | 37.04 | 10.44 |
| 91 | Irrigated  X  Rainfed | Mead, NE | 2005-04-26 | 2005-05-11 | 103 | 1093 | 18.0±0.0 | 51.19 | 58.02 | 39.55 | 11.60 |
| 92 |  | Mead, NE | 2007-05-01 | 2007-05-11 | 103 | 984 | 19.0±0.0 | 41.80 | 57.48 | 27.51 | 11.04 |
| 93 |  | Mead, NE | 2007-05-02 | 2007-05-13 | 103 | 970 | 20.0±0.0 | 38.29 | 58.12 | 26.24 | 9.94 |
| 94 |  | Mead, NE | 2009-04-22 | 2009-05-07 | 103 | 879 | - | 46.07 | 68.30 | 26.13 | 8.82 |
| 95 |  | Mead, NE | 2009-04-21 | 2009-05-05 | 103 | 876 | - | 41.52 | 48.55 | 29.72 | 10.90 |
| 96 |  | Mead, NE | 2011-05-02 | 2011-05-13 | 111 | 882 | - | 48.40 | 62.62 | 34.14 | 8.50 |
| 97 |  | Mead, NE | 2013-05-13 | 2013-05-21 | 112 | 926 | - | 48.23 | 56.28 | 30.00 | 11.03 |
| 98 |  | Mead, NE | 2013-04-30 | 2013-05-15 | 112 | 1023 | - | 47.74 | 55.65 | 25.00 | 12.43 |
