# Supplementary material for: Maize Leaf Appearance Rates: A Synthesis From the United States Corn Belt
Source: Front Plant Sci. 2022 Apr 5;13:872738. doi: 10.3389/fpls.2022.872738 (PMC9037294; doi:10.3389/fpls.2022.872738)

**Supplemental Figure S1.** Correlation analysis at different windows of growing degree days between average incident radiation and transition leaf of the bilinear model, phyllochron of the linear model, first phase phyllochron of the bilinear model, and second phase phyllochron of the bilinear model.


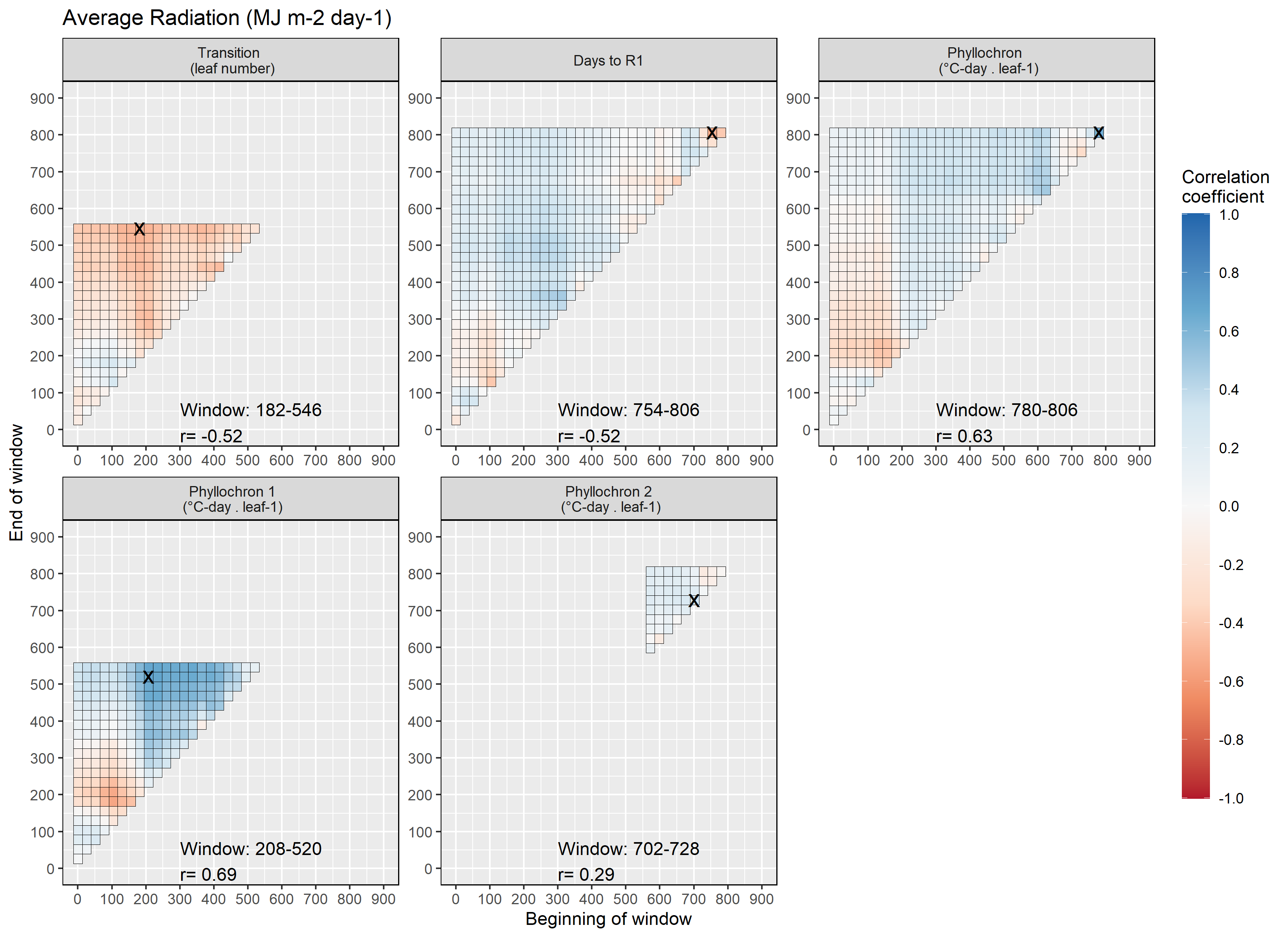


**Supplemental Figure S2.** Correlation analysis at different windows of growing degree days between average photoperiod and transition leaf of the bilinear model, phyllochron of the linear model, first phase phyllochron of the bilinear model, and second phase phyllochron of the bilinear model.


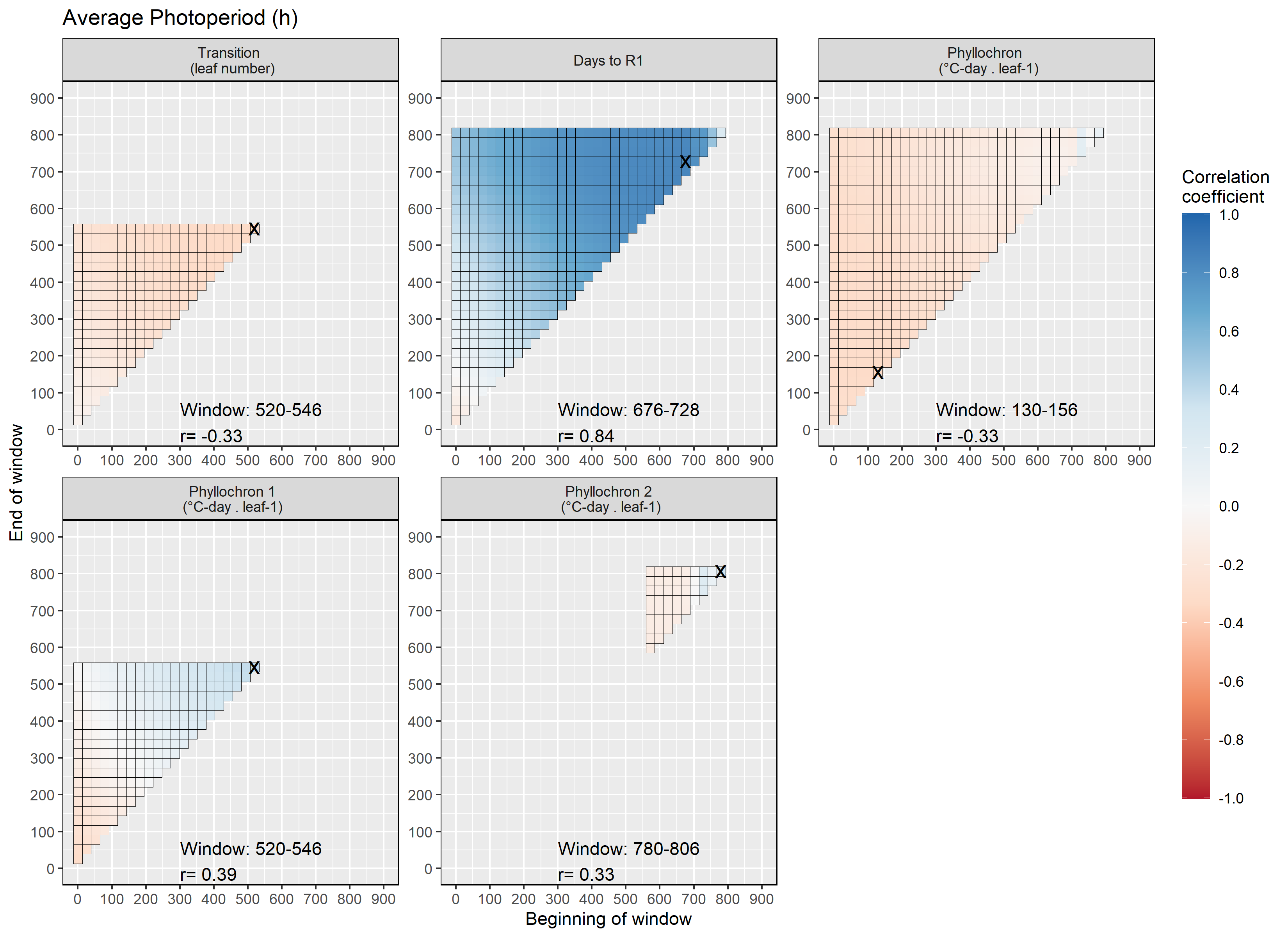


**Supplemental Figure S3.** Correlation analysis at different windows of growing degree days between average temperature and transition leaf of the bilinear model, phyllochron of the linear model, first phase phyllochron of the bilinear model, and second phase phyllochron of the bilinear model.


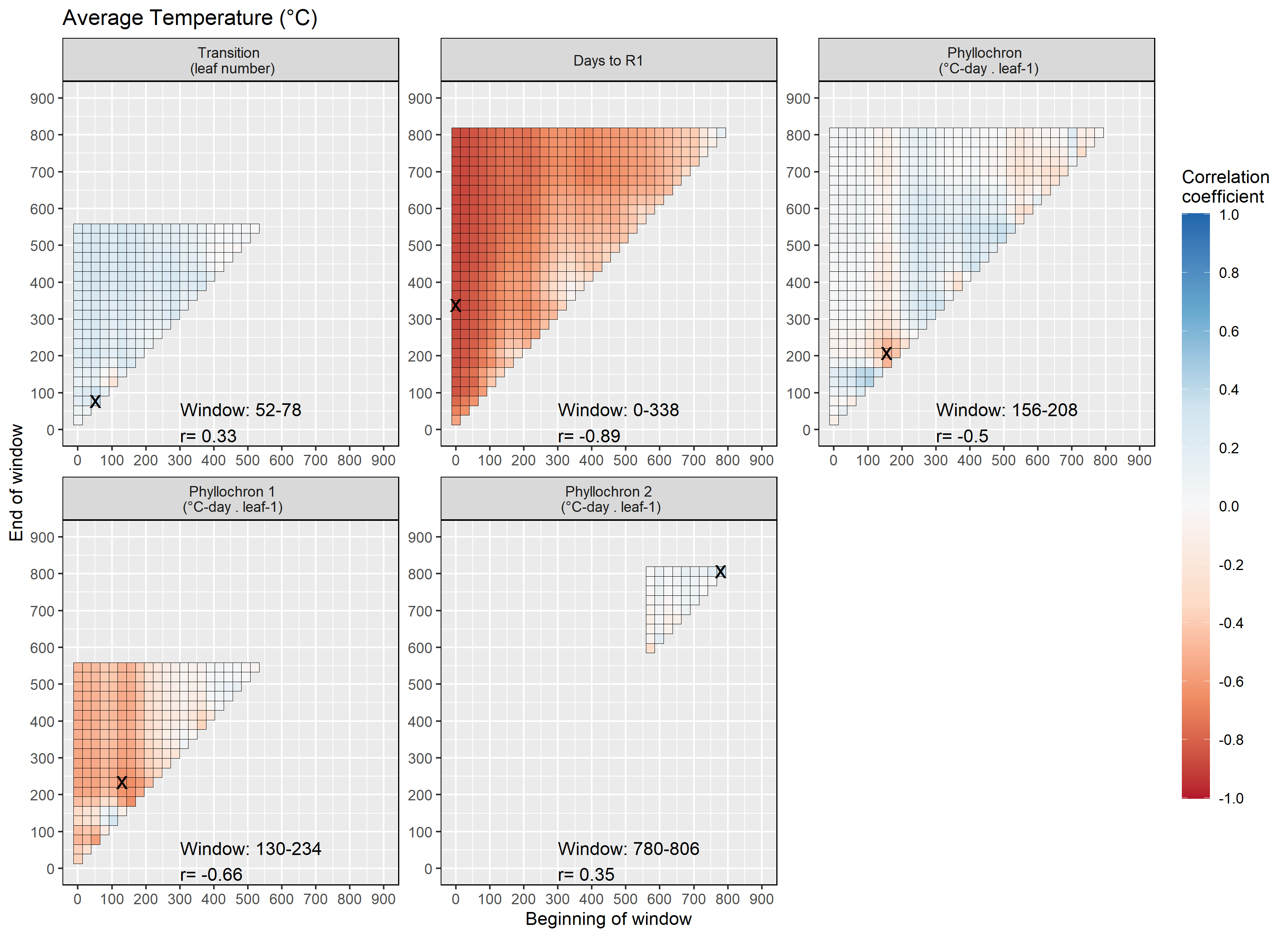


**Supplemental Figure S4.** Correlation analysis at different windows of growing degree days between cumulative rain and transition leaf of the bilinear model, phyllochron of the linear model, first phase phyllochron of the bilinear model, and second phase phyllochron of the bilinear model.
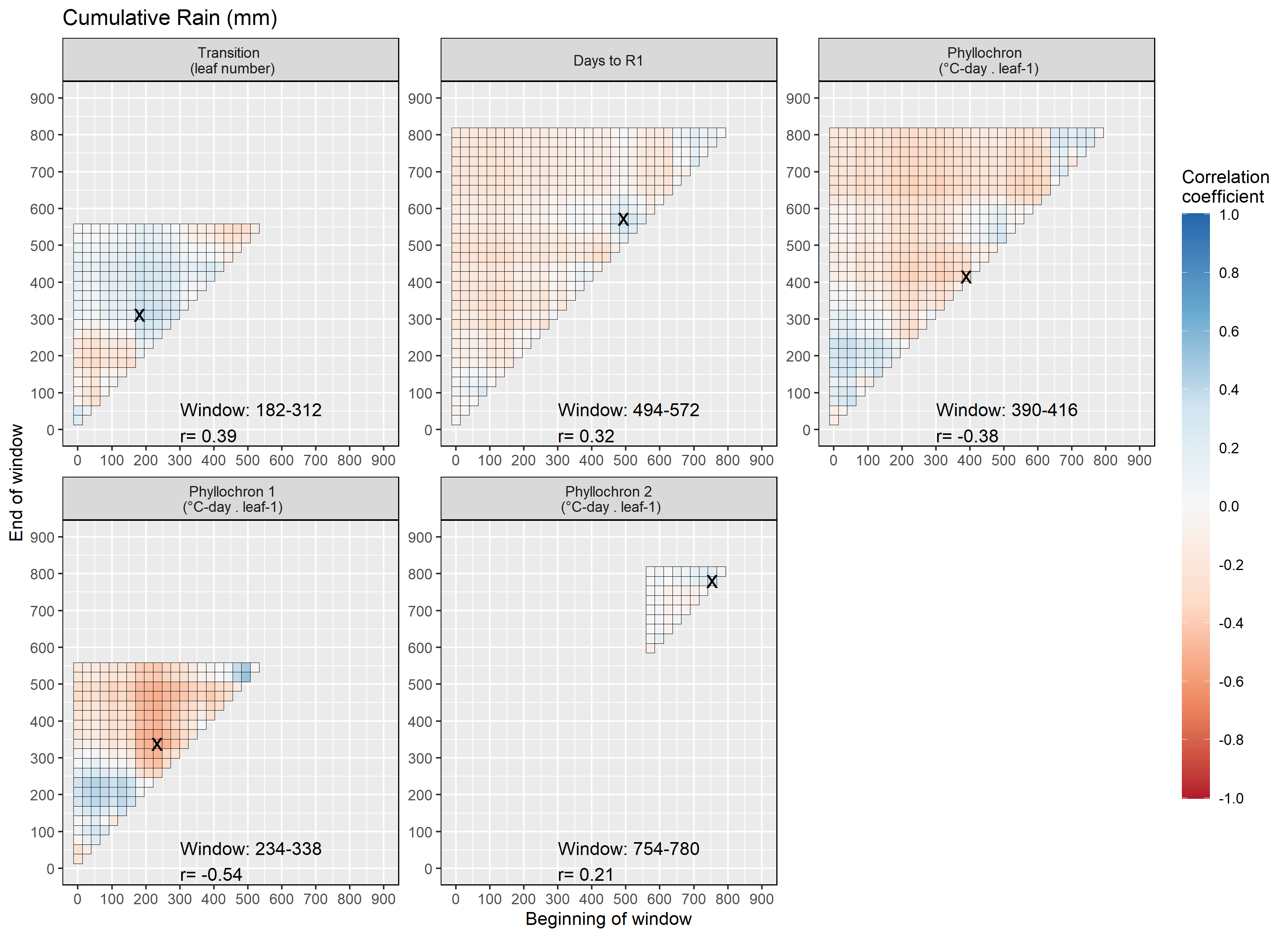


**Supplemental Figure S5.** Relationships between predicted and observed biomass (A) and leaf number (B).
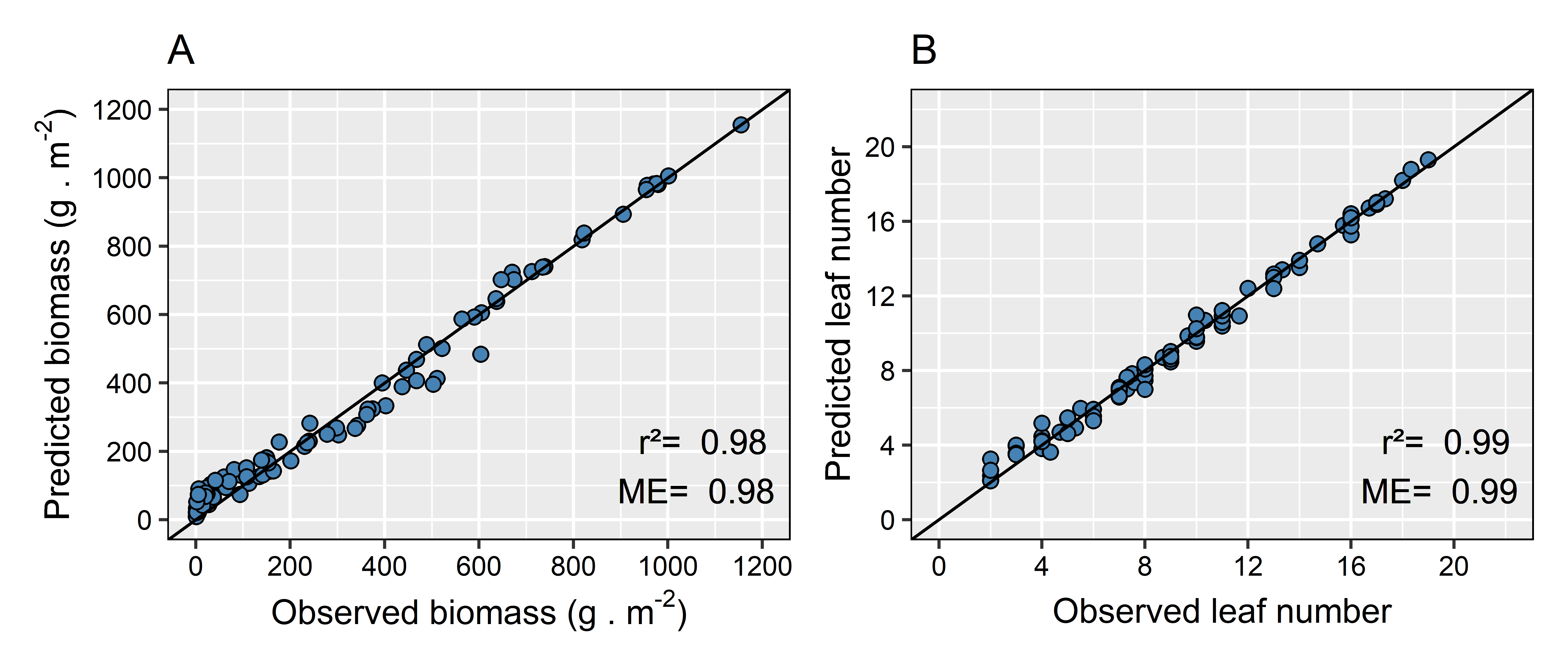

Supplement: Supplementary file 2 [file Data_Sheet_1.docx]
